# Impact of ejection fraction changes on long-term outcomes in VA-ECMO patients

**DOI:** 10.1097/MD.0000000000042306

**Published:** 2025-05-16

**Authors:** Cheng-Ta Yang, Yu-Ting Cheng, Yi-Hsin Chan, Victor Chien-Chia Wu, Dong-Yi Chen, Kuo-Chun Hung, Fu-Chih Hsiao, Ying-Chang Tung, Chia-Pin Lin, Pao-Hsien Chu, Shao-Wei Chen

**Affiliations:** a Department of Medical Education, Chang Gung University, Linkou Chang Gung Memorial Hospital, Taoyuan City, Taiwan; b Division of Thoracic and Cardiovascular Surgery, Department of Surgery, Chang Gung Memorial Hospital, Linkou Medical Center, Chang Gung University, Taoyuan City, Taiwan; c Department of Cardiology, Chang Gung Memorial Hospital, Linkou Medical Center, Chang Gung University, Taoyuan City, Taiwan; d Department of Medical Research and Development, Center for Big Data Analytics and Statistics, Chang Gung Memorial Hospital at Linkou, Taoyuan, Taiwan.

**Keywords:** ECMO, long myocardial function, term follow up, VA

## Abstract

There is limited evidence regarding the association between myocardial function requiring extracorporeal membrane oxygenation (ECMO) and long-term survival rate in patients who reach hospital discharge. This study investigates the association between myocardial function parameters collected at different times from weaning from ECMO to long-term follow-up and the long-term mortality rate. This retrospective study investigates the effect of EF timing in the long-term. A cohort of 403 patients successfully weaned from veno-arterial ECMO (VA-ECMO) was identified from 1300 patients who underwent VA-ECMO between 2003 and 2018 after applying exclusion criteria for age and indications not of interest in the Chang Gung Memorial Hospital Research Database (CGRD). The study revealed that a notable improvement in ejection fraction (EF) percentile between ECMO placement and successful weaning was significantly linked to reduced cumulative mortality as were higher EF values before discharge. However, no significant association was found between lower long-term mortality and EF change from discharge to mid-term follow-up, or the maximum EF at mid-term follow-up. Improvements in cardiac function following the use of VA-ECMO and better baseline cardiac function are associated with lower long-term mortality. The study showed that EF monitoring at ECMO insertion and before discharge can inform physicians regarding patients’ long-term outcomes. EF percentile improvement from insertion to weaning could be a positive indicator of successful weaning.

## 1. Introduction

Extracorporeal membrane oxygenation (ECMO) provides hemodynamic stability in patients with severe cardiopulmonary dysfunction. Veno-arterial ECMO (VA-ECMO) has been increasingly implemented during extracorporeal cardiopulmonary resuscitation in emergency settings as first-line circulatory support^[1]^ and as a bridge to myocardial recovery^[2]^ in cardiogenic shocks (CSs) and post-cardiotomy shocks.

By providing oxygenated blood flow to the failing heart, VA-ECMO allows hemodynamic stability and influences left ventricular function throughout the intervention. Basal left ventricle (LV) function during weaning is important in determining myocardial recovery and mid-term survival.^[3,4]^ However, the role of the dynamic changes in ejection fraction (EF) during VA-ECMO support remains to be investigated. Moreover, there is limited data on the long-term follow-up of patients who survive discharge due to high mortality and loss of follow-up. While Wu et al, previously identified a significantly worse short-to-mid-term survival in post-cardiotomy shock resuscitation with postoperative LVEF < 30% using the database from our medical center, there is no further data on patients with other indications.^[5]^ In addition, despite identifying an association between EF > 30% at weaning and significantly improved survived to discharge outcomes,^[4]^ there is no current data on a more detailed dynamic change in EF at weaning, before discharge, and during follow-up for patients with indications from post-cardiotomy shock, extracorporeal cardiopulmonary resuscitation (ECPR), and cardiogenic shock (CS). Furthermore, there is little evidence regarding the association between these parameters and long-term mortality.

We aimed to provide a comprehensive analysis of the effects of changes in myocardial function and its absolute baseline values at different time points on the long-term outcome, to improve the clinical use of EF for physicians guiding both weaning strategies and managing follow-up in patients who survive to discharge. This paper mainly focuses on VA-ECMO, myocardial function at different times, and long-term survival outcomes.

## 2. Methods

### 2.1. Data source

Study data were collected routinely from the electronic medical records of the Chang Gung Research Database (CGRD) of the Chang Gung Memorial Hospitals (CGMH) between 2003 and 2018. The CGMH system contains 3 medical centers and 4 regional hospitals with over 9000 beds and 30,000 outpatients daily, one of the largest medical systems in Asia. The multi-institutional electronic medical record database has been used for real-world retrospective studies in Taiwan, containing detailed clinical data, including claims data for insurance purposes, operation notes (free-typing text), discharge records (free-typing text), and pathological and laboratory results.^[6]^ Encrypted digital medical records have been in use for over 20 years and are unlabeled for research purposes while protecting patients’ privacy. The study received ethical and administrative approval from the Institutional Review Board of CGMH (202100124B0). Informed consent for participants was waived.

### 2.2. Study design and patient identification

**Figure 1.**
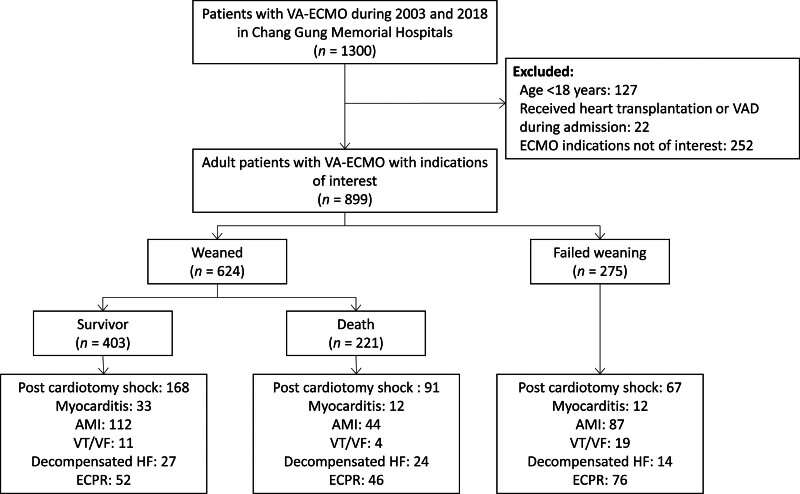
The flowchart for the inclusion and exclusion of the study patients. AMI = acute myocardial infarction, ECMO = extracorporeal membrane oxygenation, ECPR = extracorporeal cardiopulmonary resuscitation, HF = heart failure, VA = veno-arterial, VF = ventricular fibrillation, VT = ventricular tachycardia.

This was designed as a retrospective cohort study of VA-ECMO data collected between 2003 and 2018 in the CGRD. The data of VA-ECMO were ascertained by manually examining the operation notes (surgical reports) and 1300 patients administered VA-ECMO were identified. The exclusion criteria were patients under 18 years of age (n = 127), those who received a heart transplant or ventricular assist device (VAD) (n = 22) during admission, and those with indications for diseases other than primary cardiac disease (n = 252), including septic shock, pulmonary embolism, social indications, and others (Fig. 1). Indications were judged based on operation notes and discharge notes. 899 patients with indications of interest were included, including post-cardiotomy shock, myocarditis, acute myocardial infarction (AMI), ventricular tachycardia (VT) or ventricular fibrillation, decompensated heart failure, and ECPR. The remaining 899 patients were classified into the weaned (n = 624) and failed weaning (n = 275) groups. The time frame for successful weaning was set to a survival of more than 24 hours after weaning to decannulation. Among the 624 weaned patients, 403 survivors were included in further analysis of the association between LVEF, LVEF change, and mortality risk (Fig. 1).

### 2.3. extracorporeal membrane oxygenation flow rates and turn-down study

The initial mode after ECMO insertion is full support mode, with the ECMO flow target cardiac indexed at 2.0 L/min/m^2^, adjusted accordingly based on parameters such as lactate and central vein saturation. There was a turndown study during heart function recovery, the flow was gradually decreased from 1.5 to 1 L/min and then to 0.5 L/min at weaning. We kept track of relevant parameters: LVEF, aortic velocity-time integral, and lateral mitral annulus peak systolic velocity. We combined them with ECHO parameters and the inotropic score to assess the decision to remove ECMO.

### 2.4. Ejection fraction and outcome

The study outcome was all-cause mortality after discharge. Patients were followed from the day of discharge to the day of death or December 31, 2018, whichever came first. Only deaths occurring within the hospital system or among patients with out-of-hospital cardiac arrest who were brought to the facility could be identified. The exposure variables in this study were LVEF according to different definitions. The first definition was an improvement from the perioperative period (before ECMO insertion) to before discharge (available number = 102). The second definition was LVEF before discharge (available number = 238). The third definition was an improvement from discharge to the 180^th^ day after discharge (available number = 88). The fourth definition was LVEF as the 180^th^ day after discharge (available number = 177). Echocardiography closest (± 90 days) to the 180^th^ day after discharge was selected for analysis.

### 2.5. Covariates

Covariates included baseline characteristics and ECMO-related features. Baseline characteristics include demographics (age, sex, body mass index, and smoking), comorbidities (diabetes, hypertension, and 14 others), Charlson comorbidity index (CCI) total score, and preoperative laboratory data (hemoglobin, serum creatinine, and 9 others). ECMO-related features include indications, duration from admission to ECMO insertion, flow rate, turn rate, oxygen flow, peripheral/central, and perioperative treatments, including intra-aortic balloon pump, percutaneous coronary intervention (PCI), percutaneous transluminal coronary angioplasty, and coronary artery bypass graft. Comorbidities and CCI scores were extracted using the International Classification of Diseases, Ninth Revision, Clinical Modification (ICD-9-CM) before 2016, and the Tenth Revision (ICD-10-CM) in and after 2016. Laboratory data were captured at the time closest to the surgery, always within 24 hours.

### 2.6. Statistics

The baseline characteristics and ECMO-related features of the patients who died during admission vs those who survived discharge were compared using an independent sample t-test for continuous variables or the chi-square test for categorical variables. The trend of the proportions of in-hospital deaths across the study years (2003–2018) was assessed using the Cochran–Armitage test. The trend of LVEF values across ECMO insertion, before discharge, and the 180^th^ day after discharge was tested using a generalized estimating equation (with identity linking function and normal distribution), in which the analyzed cohort was restricted to the 403 patients who survived to discharge. LVEF values with the 4 definitions were categorized into 2 or 3 groups, as appropriate. The risk of all-cause mortality between the groups (e.g., LVEF before discharge: <30%, 30% to 49%, and ≥ 50%) was compared using a log-rank test. Owing to the limited sample size, no covariate adjustment was made. Two-sided *P*-values < .05 were considered statistically significant. SAS version 9.4 (SAS Institute) was used for statistical analyses.

## 3. Results

### 3.1. Epidemiological information

**Figure 2.**
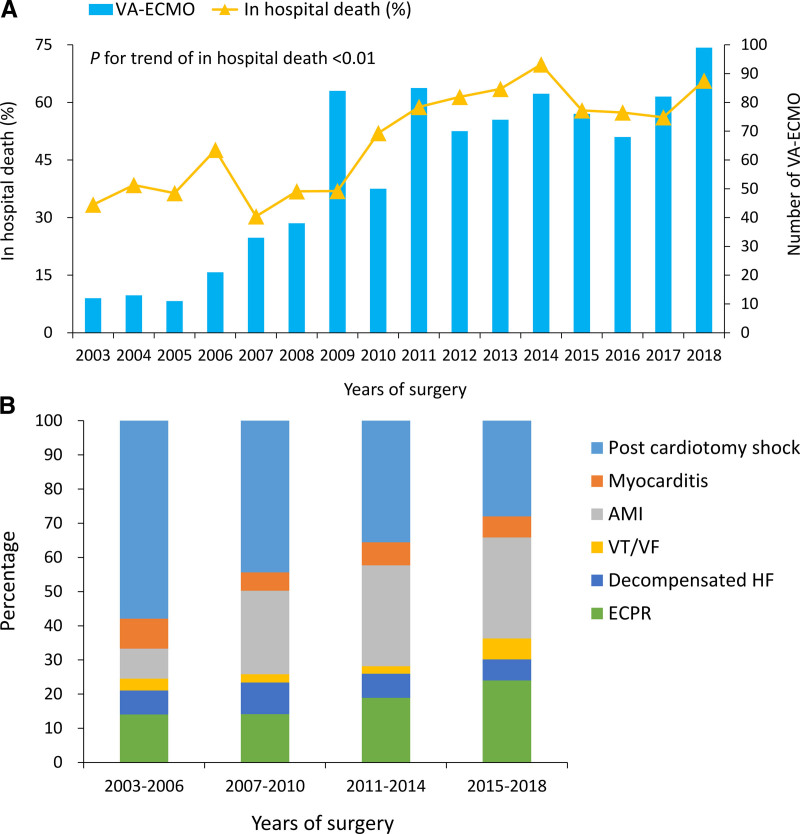
The number of VA-ECMO and proportion of in-hospital deaths across years (A) and the distribution of indications across the study period (B). AMI = acute myocardial infarction, ECMO = extracorporeal membrane oxygenation, ECPR = extracorporeal cardiopulmonary resuscitation, HF = heart failure, VA = veno-arterial, VF = ventricular fibrillation, VT = ventricular tachycardia.

The distribution of indications for patients who were weaned and survived discharge weaned and died during admission, and failed weaning are listed in Figure 1. The number of extracorporeal membrane oxygenation (ECMO) procedures and the proportion of in-hospital deaths increased with year (Fig. 2A). The proportion of post-cardiotomy shock decreased over time, while that of AMI and ECPR increased over the years (Fig. 2B).

### 3.2. Baseline characteristics

Of the 899 patients supported with VA-ECMO, the mean age was 58.7 years and 70% (n = 627) were male. 45% (n = 403) of patients survived to discharge. Compared to patients who died during admission, those who survived discharge were younger and had a lower prevalence of diabetes, coronary artery disease, peripheral artery disease, myocardial infarction, chronic kidney disease, bleeding events, and malignancy. The CCI score was also significantly lower in patients who survived to discharge. Patients who survived to discharge had lower levels of PaCO2, creatinine, liver function markers, and lactic acid but higher pH and platelet count (Table 1).

**Table 1:** Baseline characteristics of patients who underwent VA-ECMO by the survival status at discharge.

Variable	Available number	Total (n = 899)	In-hospital death (n = 496)	Survivor to discharge (n = 403)	*P*
Demographics					
Age, yr	899	58.7 ± 14.9	60.7 ± 14.8	56.2 ± 14.7	<.01
Male	899	627 (69.7)	337 (67.9)	290 (72.0)	.19
Body mass index, kg/m^2^	794	25.4 ± 4.6	25.5 ± 5.0	25.3 ± 4.1	.53
Smoking	899	324 (36.0)	178 (35.9)	146 (36.2)	.92
Comorbid conditions					
Diabetes	899	295 (32.8)	182 (36.7)	113 (28.0)	.01
Hypertension	899	472 (52.5)	276 (55.6)	196 (48.6)	.04
Chronic obstructive pulmonary disease	899	86 (9.6)	53 (10.7)	33 (8.2)	.21
Liver cirrhosis	899	29 (3.2)	18 (3.6)	11 (2.7)	.45
Coronary artery disease	899	528 (58.7)	319 (64.3)	209 (51.9)	<.01
Atrial fibrillation	899	152 (16.9)	84 (16.9)	68 (16.9)	.98
Peripheral artery disease	899	150 (16.7)	103 (20.8)	47 (11.7)	<.01
Cerebrovascular accident	899	83 (9.2)	54 (10.9)	29 (7.2)	.06
Myocardial infarction	899	195 (21.7)	128 (25.8)	67 (16.6)	<.01
Hyperlipidemia	899	268 (29.8)	149 (30.0)	119 (29.5)	.87
Chronic kidney disease	899	360 (40.0)	252 (50.8)	108 (26.8)	<.01
Gastrointestinal bleeding	899	121 (13.5)	82 (16.5)	39 (9.7)	<.01
Major bleeding	899	60 (6.7)	41 (8.3)	19 (4.7)	.03
Gout	899	90 (10.0)	57 (11.5)	33 (8.2)	.10
Malignancy	899	76 (8.5)	53 (10.7)	23 (5.7)	.01
Heart failure hospitalization	899	109 (12.1)	60 (12.1)	49 (12.2)	.98
Charlson Comorbidity Index score	899	3.2 ± 2.7	3.8 ± 2.9	2.5 ± 2.3	<.01
Preoperative laboratory data					
PaCO_2_, mm-Hg	348	38.6 ± 19.1	40.7 ± 22.0	36.2 ± 14.7	.03
PH	348	7.3 ± 0.2	7.2 ± 0.2	7.3 ± 0.2	<.01
Hemoglobin, g/dL	882	10.9 ± 2.9	10.8 ± 3.1	11.0 ± 2.6	.25
White blood cell, 10^3^/μL	874	13.6 ± 6.6	13.6 ± 7.0	13.7 ± 6.0	.79
Platelets, 10^3^/μL	877	163.5 ± 82.0	154.8 ± 81.9	173.9 ± 81.0	<.01
Creatinine, mg/dL	881	2.2 ± 2.1	2.4 ± 2.3	1.9 ± 1.9	<.01
ALT, U/L	671	69.0 (32.0–205.0)	77.0 (35.0–210.0)	57.0 (29.0–169.0)	.02
AST, U/L	742	102.5 (52.0–266.0)	117.0 (59.0–325.0)	89.0 (47.5–198.5)	<.01
Total bilirubin, mg/dL	654	1.5 ± 1.6	1.5 ± 1.8	1.4 ± 1.4	.50
Lactic acid, mg/dL	548	95.8 ± 64.9	112.6 ± 64.1	75.9 ± 60.1	<.01
Troponin I, ng/mL	675	22.5 ± 46.9	24.1 ± 51.0	20.5 ± 41.3	.32

Data were presented as frequency (percentage) or mean ± standard deviation.

ALT = alanine aminotransferase, AST = aspartate aminotransferase, ECMO = extracorporeal membrane oxygenation, PaCO2 = partial pressure of carbon dioxide, VA = veno-arterial.

### 3.3. ECMO-related features

The most common indications for ECMO were post-cardiotomy shock (36.3%), AMI (27%), ECPR (19.4%), decompensated heart failure (7.2%), myocarditis (6.3%), and VT/VF (3.8%). Compared to patients who died during admission, those who survived to discharge were more likely to have a post-cardiotomy shock (41.7% vs 31.9%), less likely to have ECPR (12.9% vs 24.6%), and had more perioperative treatment with PCI/PTCA (21.8% vs 14.9%) (Table 2).

**Table 2:** ECMO-related features of patients who underwent VA-ECMO by the survival status at discharge.

Variable	Available number	Total (n = 899)	In-hospital death (n = 496)	Survivor to discharge (n = 403)	*P*
Indication					
Post-cardiotomy shock	899	326 (36.3)	158 (31.9)	168 (41.7)	<.01
Myocarditis		57 (6.3)	24 (4.8)	33 (8.2)	.04
Acute myocardial infarction		243 (27.0)	131 (26.4)	112 (27.8)	.64
VT/ VF		34 (3.8)	23 (4.6)	11 (2.7)	.14
Decompensated heart failure		65 (7.2)	38 (7.7)	27 (6.7)	.58
ECPR		174 (19.4)	122 (24.6)	52 (12.9)	<.01
Duration from admission to ECMO insertion, day	899	4.5 ± 6.2	4.6 ± 6.6	4.4 ± 5.8	.67
Flow rate, m^3^/s	396	3281.9 ± 859.3	3250.9 ± 864.4	3328.5 ± 852.2	.38
Turn rate, units	307	2158.6 ± 577.8	2158.4 ± 598.9	2158.9 ± 547.2	.99
Oxygen flow, units	78	4049.6 ± 793.3	3999.3 ± 902.3	4121.9 ± 610.0	.51
Peripheral/ central					
Peripheral	899	878 (97.7)	483 (97.4)	395 (98.0)	.53
Central		21 (2.3)	13 (2.6)	8 (2.0)	
Treatment before, during or after ECMO					
IABP	899	346 (38.5)	179 (36.1)	167 (41.4)	.10
PCI/ PTCA	899	162 (18.0)	74 (14.9)	88 (21.8)	.01
CABG	899	193 (21.5)	102 (20.6)	91 (22.6)	.46

Data were presented as frequency (percentage) or mean ± standard deviation.

CABG = coronary artery bypass graft, ECMO = extracorporeal membrane oxygenation, ECPR = extracorporeal cardiopulmonary resuscitation, IABP = intra-aortic balloon pump, PCI = percutaneous coronary intervention, PTCA = percutaneous transluminal coronary angioplasty, VA = veno-arterial, VF = ventricular fibrillation, VT = ventricular tachycardia.

### 3.4. LVEF and all-cause mortality

**Figure 3.**
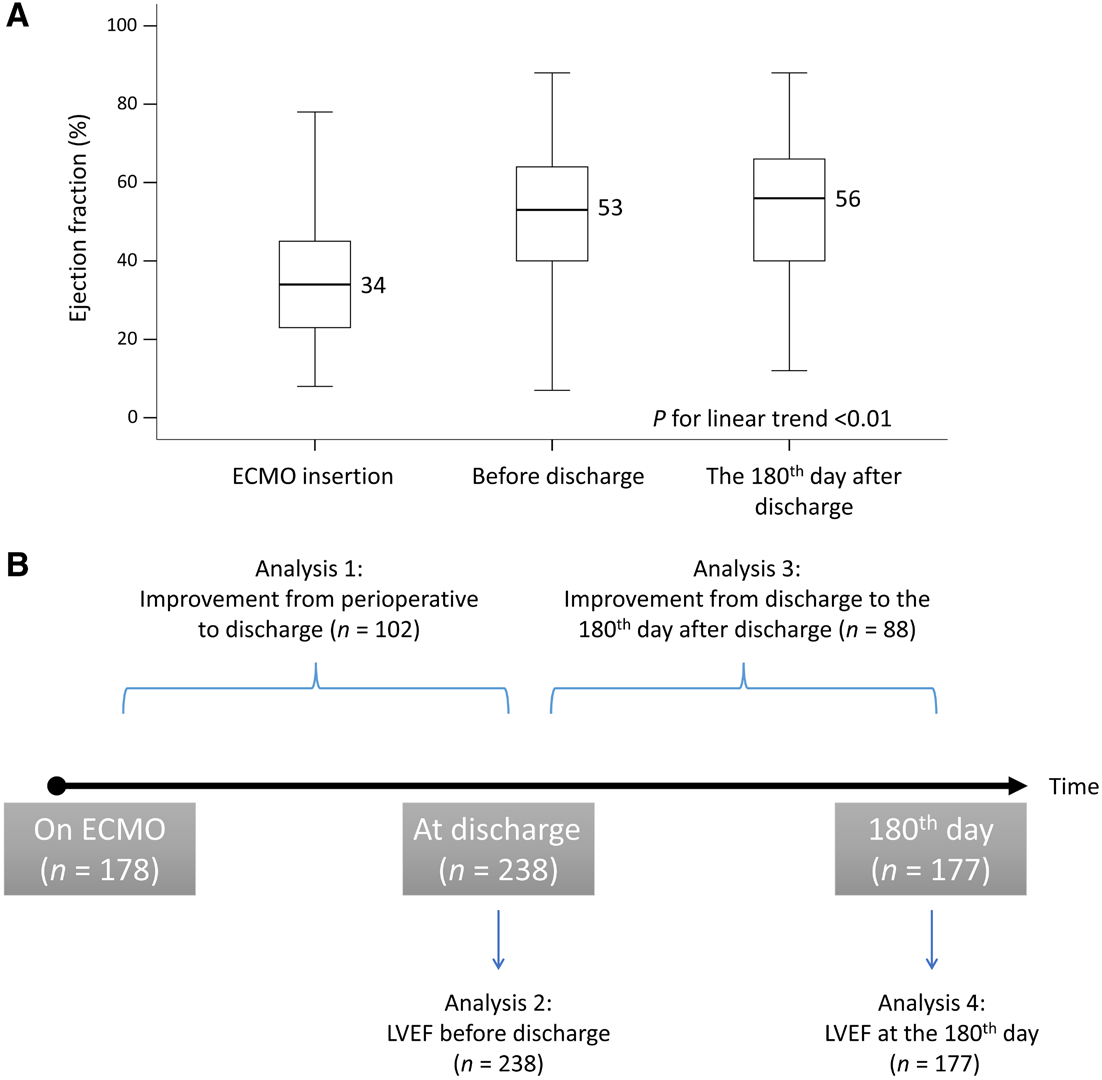
The LVEF values at ECMO insertion, before discharge and the 180th day after discharge (A) and the analytic framework of the study (B). ECMO = extracorporeal membrane oxygenation, LVEF = left ventricular ejection fraction.

**Figure 4.**
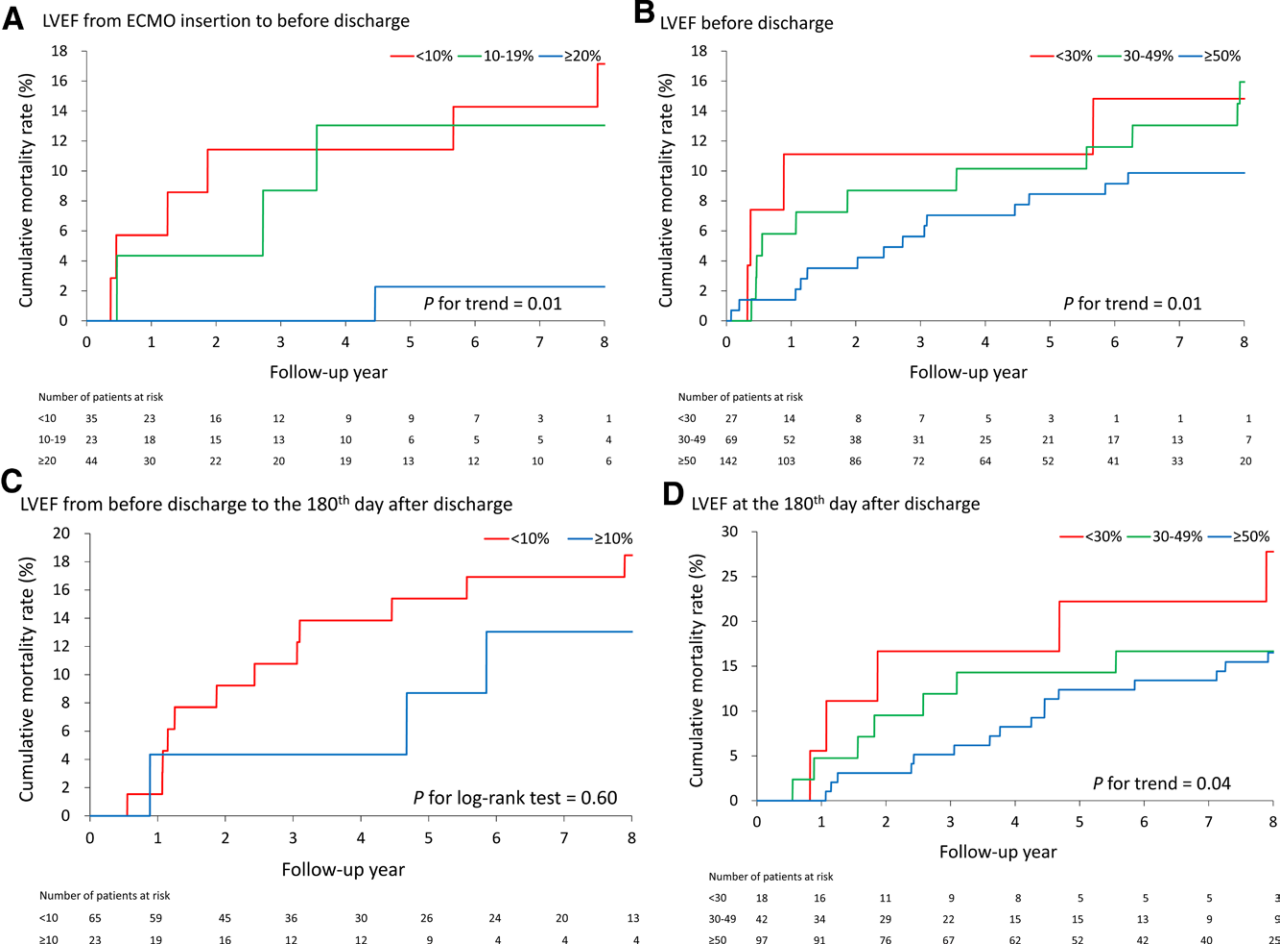
The relationship between risk of all-cause mortality and LVEF with different definitions: the improvement from ECMO insertion to before discharge (A), the LVEF value before discharge (B), before discharge to the 180th day after discharge (C), and LVEF at the 180th day after discharge (D). ECMO = extracorporeal membrane oxygenation, LVEF = left ventricular ejection fraction.

The results showed that LVEF significantly improved from ECMO insertion (median: 34%) to before discharge (median: 53%) and the 180^th^ day after discharge (median: 56%) (*P* for trend < .01; Fig. 3A). According to the analytical framework (Fig. 3B), the first analysis showed that the improvement in LVEF from ECMO insertion to before discharge was significantly correlated with a lower mortality rate (*P* for trend < .01; Fig. 4A). The second analysis demonstrated that a greater LVEF was significantly correlated with a lower mortality rate (*P* for trend < .01; Fig. 4B). The third analysis revealed that the mortality risk of patients who improved (≥10%, n = 25) from before discharge to the 180^th^ day after discharge vs those who did not improve (<10%) was not significantly different (*P* = .60; Fig. 4C). Finally, the fourth analysis indicated that a greater LVEF on the 180^th^ day after discharge was significantly correlated with a lower mortality rate (*P* for trend = .04; Fig. 4D).

## 4. Discussion

In this multicenter analysis study, we retrospectively evaluated patients with CS supported by VA-ECMO who successfully survived hospital discharge. To date, this was the largest multicenter observational retrospective study of myocardial function in VA-ECMO patients with CS. This was also the first study to provide insights into patients who survived discharge with their cardiac function data at admission, before discharge, and long-term follow-up with survival analysis. We found that percentile improvement in EF between ECMO placement and successful weaning was significantly associated with lower cumulative mortality. Second, the EF value before discharge was significantly associated with better survival. Lastly, the association of low long-term mortality with EF change from discharge to mid-term follow-up and the maximum EF at mid-term follow-up was found to be nonsignificant.

VA-ECMO acts as a temporary bridge^[7,8]^ for myocardial function recovery or further treatment.^[9–12]^ Post-cardiotomy shock develops in around 1% of adult patients who require temporal circulatory mechanical support during or after cardiac surgery with a 63.3% successful weaning rate and 24.8% discharge rate.^[13,14]^ Chen et al, previously showed that despite better outcomes in the first year, compared to non-ECMO groups, the ECMO group showed similar outcomes after first year follow-up in patients with post-cardiotomy shock using the same database in our hospital.^[14]^ In our study, the most common indication for VA-ECMO was a post-cardiotomy shock, and those who survived until discharge were more likely to have a post-cardiotomy shock as an indication. (41.7% vs 31.9%). This finding is in line with past research.^[14–16]^

Echocardiography is crucial in weaning trials and the assessment of cardiac function recovery.^[17,18]^ While most studies focused on identifying mortality predictors at weaning or discharge, evidence of the short-term and long-term outcomes of VA-ECMO remained limited.

In another retrospective study using data from our hospital center, a preoperative LVEF > 30% was associated with a lower long-term cumulative mortality rate in patients receiving ECMO for post-cardiotomy shock.^[5]^ Although Sertic et al, further showed that severe LV dysfunction (LVEF < 30%) was identified to be associated with a lower postweaning survival rate, it was limited to the absolute value of LVEF.^[4]^ Our study was the first to investigate dynamic changes in LV function during ECMO support and from discharge to mid-term follow-up. We showed an overall improvement in LVEF in patients who survived mid-term follow-up. The major findings of our study suggested that improvements in ejection fraction were strongly associated with favorable long-term survival. An improvement in EF may indicate restored myocardial function. It could serve as a direct monitoring parameter in complex weaning strategies,^[3]^ and for further treatment since these patients have better long-term survival.

Our study provided further evidence of the impact of EF on long-term survival at different time points. We confirmed the results of past studies that an absolute value of EF < 30% before discharge is associated with worse outcomes and found that the EF > 50% group fared even better in first-year survival.^[4,5]^ However, a higher absolute value of the maximum EF at 6 months follow-up was not significantly associated with reduced mortality. Improvements in EF from discharge to 6 months also showed no significance. One possible explanation was that improvement in EF from ECMO placement to before discharge or higher EF at weaning played a more decisive role in deciding mortality, reducing the effect seen on future improvements in EF. Another possibility was that the smaller follow-up group size failed to show significance. It is worth noting that percentile improvement of LVEF during VA-ECMO insertion cannot be inferred as the cause of improved long-term mortality, since worse long-term mortality has also been shown to be associated with other predictive factors, such as indications, duration of VA-ECMO support, diabetes, and previous myocardial infarction.^[4,19]^

The clinical implications of our findings were 3-fold. *Weaning protocol:* Additional parameters, such as LVEF, aortic velocity-time integral, and lateral mitral annulus peak systolic velocity, should be monitored during the weaning process. Notable improvements in the EF percentile > 20%) between ECMO placement and weaning could be a positive indicator, increasing confidence in successful weaning. *Discharge planning:* Patients with low EF at weaning were at higher risk of mortality, necessitating referrals to heart failure specialists and cardiac rehabilitation programs to optimize outcomes. *Post-discharge monitoring:* Early enrollment in heart transplant programs or elective durable VAD therapy could be recommended for select candidates. Close monitoring and follow-up of discharged patients were essential to identify individuals at risk of first-year mortality and to facilitate timely decisions regarding advanced therapies, such as VADs or heart transplants.^[7,20]^

### 4.1. Limitations

This study had several limitations. First, limitations common to its retrospective and observational nature may be relevant, including the possibility of a selection bias. Further analyses are required for patients bridged to VADs and heart transplants and for those with indications not of interest. Confounding factors that could affect survival other than directly changing myocardial function render the relationship between survival and EF relative rather than causative. Second, the patient group size in our series could have affected the significance of our results, especially in the analyses involving missing mid-term and long-term EF data due to loss of follow-up or changes in electronic records. Finally, there is a limitation in extending the results to other countries owing to the higher ECPR usage in Taiwan,^[21]^ which lacks records of baseline EF at presentation to the emergency room and lower numbers of VADs and heart transplants.

## 5. Conclusion

Ejection fraction monitoring at ECMO insertion and before discharge can inform physicians regarding patients’ long-term outcomes. EF percentile improvement from insertion to hospital discharge and a higher absolute EF value before discharge were associated with lower long-term mortality rates. Studies of other cardiac echocardiography parameters using long-term follow-up data should be performed in future research to validate these findings.

## Acknowledgments

The authors thank the statistical assistance and wish to acknowledge the support of the Maintenance Project of the Center for Big Data Analytics and Statistics, Chang Gung Memorial Hospital, Linkou for study design and monitoring, data analysis, and interpretation. The authors also thank Alfred Hsing-Fen Lin and Ben Yu-Lin Chou for their assistance with the statistical analysis.

## Author contributions

**Conceptualization:** Yu-Ting Cheng, Shao-Wei Chen.

**Data curation:** Cheng-Ta Yang, Yu-Ting Cheng.

**Funding acquisition:** Shao-Wei Chen.

**Investigation:** Cheng-Ta Yang, Yu-Ting Cheng, Shao-Wei Chen.

**Methodology:** Yu-Ting Cheng, Shao-Wei Chen.

**Resources:** Shao-Wei Chen.

**Supervision:** Yu-Ting Cheng, Yi‐Hsin Chan.

**Writing – original draft:** Cheng-Ta Yang.

**Writing – review & editing:** Cheng-Ta Yang, Yu-Ting Cheng, Yi‐Hsin Chan, Victor Chien-Chia Wu, Dong-Yi Chen, Kuo-Chun Hung, Fu-Chih Hsiao, Ying-Chang Tung, Chia-Pin Lin, Pao-Hsien Chu, Shao-Wei Chen.
